# Editorial – The future depends on how we choose to behave

**DOI:** 10.1017/ehs.2020.20

**Published:** 2020-05-08

**Authors:** Ruth Mace

In my last editorial I grumbled about 2019 and hoped for a better 2020. Well that did not happen. None of us imagined just how bad 2020 was going to be. We prepare for nuclear wars with huge investments but, it seems, not for pandemics. A few Far Eastern countries with experience of SARS seem to have mounted coordinated responses in the early days. The numbers reported from Wuhan were not correct, but as the main effect became clearer, China's experience gave the world a good six weeks to prepare; however, the warning was largely ignored.

A plethora of epidemiological models were quickly developed to predict the grim spread of covid-19, all of which highlighted the need for good data to test both their assumptions and predictions. Observed death rates could either be the result of high levels of mostly asymptomatic infection, high levels of immunity in the population, and lower mortality per infection (perhaps as low as 1 or 2/1000 cases), or this virus has lower infection rates but with higher case mortality (as high as 1/100 or even higher?) (e.g. Ferguson et al., 2020; Lourenco et al. 2020). We all hope for the former scenario, as the more immunity there is in the population, the easier it is for the disease to be eradicated (Britton, 2020; Panovska-Griffiths, 2020). However, a paucity of the relevant data on this new disease means that nobody yet knows enough to distinguish the different hypotheses. The predicted scenarios can help scare governments into action, and help them compare hypothetical outcomes; but these models are not designed to predict behaviour change.

In England, estimates suggest that covid-19 deaths peaked around April 8 (possibly earlier in London), meaning that infections could have peaked three weeks earlier (Oke and Henegan 2020). That suggests, after weeks of largely ignoring the threat, we suddenly started to change our behaviour (staying home, shunning public transport, washing our hands – and of course filling our freezers and bathroom cabinets) even before the official lockdown on 23 March. The future depends on how we choose to behave.

Evolutionary human scientists cannot necessarily predict the future either, but we can add understanding of our biological, cultural and behavioural responses to the clear and present danger that covid-19 presents. We have a long tradition in behavioural evolutionary studies of highlighting how and why differential access to resources, wealth and status influences our health, behaviour and life history across a wide range of contexts. This can help throw light on why some sections of the population are unlikely to be interested in public health advice on corona virus, or indeed in other domains. Evolutionary scientists think a lot about cooperation and when we do and do not cooperate, and why we may not necessarily be interested in the public good. How we respond to risks is also informed by life history theory. Changing our norms, including the much wider adoption of some behaviours now common mainly in East Asian countries, will surely happen soon. Changing norms is cultural evolution; and models of behavioural contagion may end up being just as relevant as their virological counterparts. Social networks with 1 or 0 nodes may prevent disease transmission, but they are not normal for a species that evolved in connected family groups, and that aspect of our evolutionary history has influenced so much of our psychology and life history. The politics, economics and genetic footprints of former plagues can inform us too. Anthropologists can help explain why ‘Western’ policies will not even be relevant in many parts of the world (especially where having three weeks’ worth of food in the fridge is not an option). We might learn a lot about tackling outbreaks from some of the world's poorest, given their experience of living with fatal infectious diseases without much prospect of medical help. The pandemic is putting these effects into sharp relief. Needless to say, *EHS* welcomes any papers on covid-19 and especially our responses to it. Non-corona science is having a hard time competing for attention at present. However, as we get over the initial shock, in addition to work on covid-19, at least some of us are returning to other scientific endeavours too.

Being reliant on internationalism, and gaining so much from meeting in groups, means that academia could be feeling the repercussions of social distancing for some time. Whilst many of us are in that blessed category of those who can work from home, that covers a range of scenarios: some of us are caring for children or volunteering to help others, while others can be very academically productive in lockdown (there are rumours that some journal editors are noticing fewer paper submissions from women, suggesting that more women fall into the former category – I hope submissions to *EHS* can put that rumour to bed, but watch this space). Fieldwork is always difficult but may be impossible for a while. We can focus our research on modelling, online experiments and analysis of existing data for now, but I worry that can only take us so far without being nourished by new data from real-world populations. The journal is doing fine. We aim to keep processing your papers within our usual timeframe, but bear with us if there are a few snags along the way.

We all missed the chance to meet at the EHBEA conference in Krakow in April, which is now postponed until March 2021. As the eye of the hurricane has moved from east to west, I am touched by the concern of my Chinese friends and collaborators and so grateful to those who have sent me face masks. Let us hope that international collaborations and scientific communities can survive current travel restrictions, hardships and xenophobia.

Will we ever get back to the *status quo ante*? Do we even want to? Will the silver lining be that we decide to pollute less and care for each other more? If no vaccine is found, there is little doubt that life will change. If our scientific colleagues do find a vaccine, that will be our lifeline, although I, for one, predict that it will not then be long before we slip back into old habits. I hope that I am proved wrong.
